# Impact of Asynchronous Electronic Communication–Based Visits on Clinical Outcomes and Health Care Delivery: Systematic Review

**DOI:** 10.2196/27531

**Published:** 2021-05-05

**Authors:** Oliver T Nguyen, Amir Alishahi Tabriz, Jinhai Huo, Karim Hanna, Christopher M Shea, Kea Turner

**Affiliations:** 1 Department of Health Outcomes and Biomedical Informatics University of Florida Gainesville, FL United States; 2 Department of Health Services Administration University of Alabama at Birmingham Birmingham, AL United States; 3 Department of Health Outcomes and Behavior Moffitt Cancer Center Tampa, FL United States; 4 Department of Oncological Sciences University of South Florida Tampa, FL United States; 5 Department of Family Medicine Morsani College of Medicine University of South Florida Tampa, FL United States; 6 Department of Health Policy and Management University of North Carolina at Chapel Hill Chapel Hill, NC United States

**Keywords:** telemedicine, telehealth, e-visits, electronic visits, digital care, outcome, delivery, review, access, utilization, cost, patient portal, eHealth

## Abstract

**Background:**

Electronic visits (e-visits) involve asynchronous communication between clinicians and patients through a secure web-based platform, such as a patient portal, to elicit symptoms and determine a diagnosis and treatment plan. E-visits are now reimbursable through Medicare due to the COVID-19 pandemic. The state of evidence regarding e-visits, such as the impact on clinical outcomes and health care delivery, is unclear.

**Objective:**

To address this gap, we examine how e-visits have impacted clinical outcomes and health care quality, access, utilization, and costs.

**Methods:**

We conducted a systematic review; MEDLINE, Embase, and Web of Science were searched from January 2000 through October 2020 for peer-reviewed studies that assessed e-visits’ impacts on clinical and health care delivery outcomes.

**Results:**

Out of 1859 papers, 19 met the inclusion criteria. E-visit usage was associated with improved or comparable clinical outcomes, especially for chronic disease management (eg, diabetes care, blood pressure management). The impact on quality of care varied across conditions. Quality of care was equivalent or better for chronic conditions, but variable quality was observed in infection management (eg, appropriate antibiotic prescribing). Similarly, the impact on health care utilization varied across conditions (eg, lower utilization for dermatology but mixed impact in primary care). Health care costs were lower for e-visits than those for in-person visits for a wide range of conditions (eg, dermatology and acute visits). No studies examined the impact of e-visits on health care access. It is difficult to draw firm conclusions about effectiveness or impact on care delivery from the studies that were included because many used observational designs.

**Conclusions:**

Overall, the evidence suggests e-visits may provide clinical outcomes that are comparable to those provided by in-person care and reduce health care costs for certain health care conditions. At the same time, there is mixed evidence on health care quality, especially regarding infection management (eg, sinusitis, urinary tract infections, conjunctivitis). Further studies are needed to test implementation strategies that might improve delivery (eg, clinical decision support for antibiotic prescribing) and to assess which conditions can be managed via e-visits.

## Introduction

Telemedicine—or the delivery of health care at a distance—can improve health care access and quality while reducing health care utilization and costs [1]. For instance, telemedicine can improve access to specialists for patients in rural areas or in underresourced care settings by increasing the convenience and availability of health care (eg, extended hours, decreased wait times) [2-4]. Studies have also demonstrated that telemedicine may achieve comparable clinical outcomes to in-person care across a variety of conditions, such as stroke care [5], heart failure [6,7], hepatitis C [8], and diabetes [8]. Telemedicine can also reduce the utilization of in-person care and reduce health care costs [3,4,9]. Furthermore, some telemedicine types have demonstrated cost-effectiveness [10,11]. Studies have shown that the impact of telemedicine on health care delivery and patient outcomes varies across telemedicine types. Some forms of telemedicine, such as telestroke [12-14], have a strong evidence base while other forms of telemedicine, such as electronic visits (e-visits), are understudied.

E-visits involve asynchronous communication between clinicians and patients through a secure web-based platform, such as a patient portal. Generally, patients answer questions about their medical history and symptoms through a structured questionnaire and upload photos (if relevant). The data are then reviewed by a clinician, who develops a diagnosis and treatment plan. Although e-visits often involve clinicians who the patient is familiar with, they can also involve a third-party clinician through direct-to-consumer telemedicine. E-visits offer notable benefits, such as allowing patients and clinicians to communicate at a convenient time (ie, eliminating scheduling barriers) and improving documentation of patient-clinician communication (eg, patients can review clinician’s instructions) [15,16]. However, e-visits have a number of implementation barriers, such as those regarding workflow integration (eg, having dedicated clinician time to respond to messages) [16-18], those regarding lack of reimbursement [15,16,19,20], and concerns about the quality of communication from patients (eg, failing to submit sufficient information for diagnosis) [21]. Health care organizations have also raised concerns about the quality of care provided through e-visits, such as the potential for inappropriate antibiotic prescribing and difficulties with providing care without being able to see the patient face-to-face [22-29]. Despite these challenges, studies have reported positive effects of e-visits on health care delivery, such as lower costs [24], comparable follow-up rates to those of in-person care (ie, a proxy for diagnostic accuracy) [30], and comparable or improved patient outcomes (eg, lower uric acid level for patients with gout) [31].

Because of COVID-19, e-visits are being implemented more frequently and are now reimbursable by Medicare and other payers in the United States [32,33]. As the nation moves forward in telemedicine implementation and health care systems decide whether to integrate telemedicine into future care delivery, it is critical to determine the costs and benefits of telemedicine models, such as e-visits. To date, a systematic review has not been conducted to assess the state of the evidence regarding e-visits. To address this gap, the objective of this review is to summarize the findings of studies examining the association of e-visits with clinical outcomes, quality of care, access to care, utilization, and costs.

## Methods

We conducted a systematic review based on PRISMA (Preferred Reporting Items for Systematic Reviews and Meta-analysis) guidelines [34].

### Data Sources and Searches

MEDLINE, Embase, and Web of Science were searched to locate peer-reviewed studies published from January 2000 through October 2020. The start date for the search was chosen because electronic visits were developed recently, and the authors did not anticipate any studies published prior to 2000 (when web-based, patient portal studies emerged). A preliminary search was done to confirm this. Studies were limited to those in the United States as systemic factors, such as reimbursement, may influence the results. All reference lists for included studies were cross-searched. Duplicate studies were removed. Multimedia Appendix 1 lists the search terms used for this search after consultation with a health sciences librarian at the University of Florida.

### Study Selection

Two reviewers (KT, OTN) independently screened papers identified from the search strategy, and assessed each for inclusion eligibility using a spreadsheet (Excel 2013, Microsoft Inc). Any discrepancy encountered was discussed until a consensus was reached.

E-visits were defined as any asynchronous electronic visit where a clinician assesses a patient’s health status, makes a diagnosis, and develops a treatment plan via a secure messaging system (eg, patient portal) [24,35]. This definition was based on how recent studies have defined e-visits [24,35]. Studies that defined an e-visit differently were excluded (eg, defines e-visit as real-time, 2-way communication). Studies were included that reported on the impact of e-visits on clinical outcomes, health care quality, access, utilization or costs. Included studies also had to be written in English, empirical (ie, reporting original research), quantitative, and peer reviewed.

### Data Extraction and Quality Assessment

For each included study, 2 investigators (KT, OTN) noted study design, outcome measurements, care setting, medical conditions studied, sample size, and major findings. Since most of the studies included were observational, the authors used the Risk of Bias Assessment Tool for Nonrandomized Studies to assess criteria specific to observational studies [36]. A *P*-value <.05 was considered significant.

### Data Synthesis and Analysis

Due to the heterogeneity of outcome measures used in our included studies, it was infeasible to conduct a meta-analysis. Consequently, findings were qualitatively grouped by outcome type (eg, clinical outcomes, costs).

## Results

**Figure 1:**
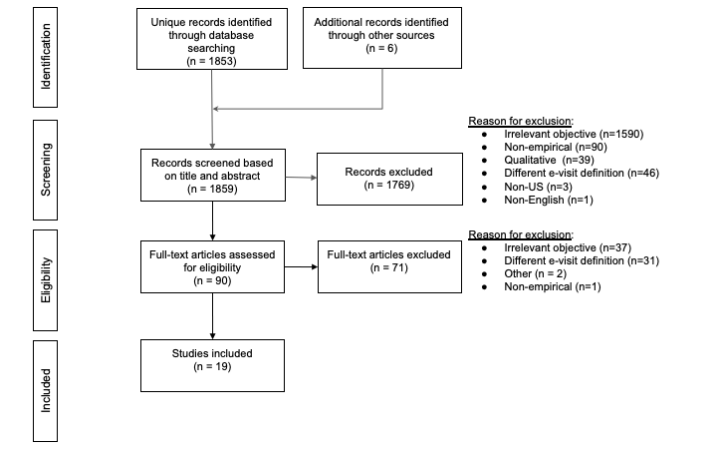
PRISMA [34] flowchart.

After reviewing 1859 studies, a total of 19 studies met our inclusion criteria. Figure 1 shows our study selection process.

### Study Characteristics

Most studies assessed clinical outcomes, quality of care, health care utilization, and costs. None reported access outcomes. Most studies were observational in design, with one study using a randomized experimental design, another study using a case study design, and another 2 studies employing quasi-experimental designs. Sixteen studies used cross-sectional or pooled cross-sectional data. Three studies used a panel design. Table 1 summarizes the study characteristics of included studies, and Table 2 summarizes findings with respect to clinical outcomes, quality of care, access to care, health care utilization, and costs.

**Table 1:** Study characteristics.

Citation	Types of health system measure	Entity provisioning e-visits	Care setting	Medical conditions treated	Sample size, n
Adamson et al [19]	Costs, utilization	Health care organization	Outpatient clinic, academic medical center	Sinusitis, depression, back pain, cough, anxiety, hypertension, abdominal pain, headache, urinary tract infections, influenza, allergic rhinitis, dermatitis, attention-deficit/hyperactive disorder, gastroesophageal reflux disease, vaginitis, upper respiratory infection, insomnia, asthma, contraception, hyperlipidemia	2531
Rohrer et al [37]	Costs, utilization	Health care organization	Outpatient clinic	Conjunctivitis, sore throat, viral illness, bronchitis, cough	390
Watson et al [38]	Clinical outcomes	Health care organization	Outpatient clinic	Acne	121
Albert et al [39]	Costs, utilization	Health care organization	Outpatient clinic, academic medical center	Blood pressure management, fractures, diabetes management, skin conditions, prostatitis, pain, sleep issues, vomiting, mononucleosis, hemorrhoids, cold symptoms	121
Courneya et al [40]	Quality of care, costs, utilization	Insurer	N/A^a^	Acute sinusitis, chronic sinusitis, urinary tract infections, conjunctivitis, viral upper respiratory infection, lower genitourinary system infection, yeast infection, otolaryngology diseases, acne, allergic rhinitis, acute bronchitis	Reported as more than 40,000
Mehrotra et al [24]	Quality of care, costs, utilization	Health care organization	Outpatient clinic, academic medical center	Sinusitis, urinary tract infections	574
North et al [41]	Costs, utilization	Health care organization	Outpatient clinic, academic medical center	No specific conditions studied, but e-visit content mentioning chest pain and respiratory complications were monitored	892
Heyworth et al [42]	Quality of care	Health care organization	Inpatient, Veterans Affairs	Diabetes, hypertension, hyperlipidemia, heart disease, prior history of myocardial infarction or stroke	51
Pathipati et al [43]	Costs, utilization	Health care organization	Outpatient clinic, academic medical center	Rash, acne, other unspecified dermatological conditions	38
Hawes et al [44]	Clinical outcomes, quality of care	Health care organization	Outpatient clinic, academic medical center	Diabetes, anticoagulation management	36
Levine et al [45]	Clinical outcomes, costs, utilization	Health care organization	Outpatient clinic, academic medical center	Hypertension	1786
Penza et al [46]	Quality of care, costs, utilization	Health care organization	Outpatient clinic, academic medical center	Conjunctivitis	505
Penza et al [47]	Clinical outcomes, costs, utilization	Health care organization	Outpatient clinic, academic medical center	Allergies, upper respiratory infection, cold sores, influenza, lice, conjunctivitis, sinusitis, sore throat, sunburn, tick exposure, urinary tract infections, yeast infection	1009
Player et al [48]	Costs, utilization	Health care organization	Outpatient clinic, academic medical center	Athlete’s foot, allergic skin reaction, eczema, cold sore, shingles rash, marine animal string, jock itch, nosebleed, poison ivy, rash, red eye, ringworm, scabies, hemorrhoids, sunburn, back pain, gout, heartburn, seasonal allergies, travel precaution, prescription refills, sexually transmitted infections, diarrhea, influenza, sinus problems, urinary problems, vaginal irritation or discharge	1565
Rajda et al [49]	Costs, utilization	Direct-to-consumer company	N/A	Acne vulgaris, atopic dermatitis, onychomycosis, psoriasis vulgaris, rosacea	395
Hertzog et al [30]	Costs, utilization	Health care organization	Outpatient clinic, academic medical center	Yeast infection, acne, allergic rhinitis, urinary tract infections, upper respiratory infection, conjunctivitis, oral sores, irritable bowel syndrome, tobacco cessation	2691
Murray et al [50]	Clinical outcomes, quality of care	Health care organization	Outpatient clinic, academic medical center	Urinary tract infections	300
Penza et al [51]	Clinical outcomes, quality of care	Health care organization	Outpatient clinic, academic medical center	Acute sinusitis	300
Yokose et al [31]	Quality of care	Health care organization	Outpatient clinic, academic medical center	Gout	124

^a^N/A: not applicable.

**Table 2:** Effects of asynchronous e-visits on clinical outcomes, quality, utilization, and costs.

Outcome type and citations		Specific measures	Impact
**Clinical outcomes**			
	Hawes et al [44]	Abnormal international normalized ratio	E-visits were associated with lower abnormal international normalized ratios than in-person visits (*P*<.05).
	Hawes et al [44]	HbA_1c_^a^	E-visits were associated with lower HbA_1c_ values than in-person visits (*P*<.001).
	Hawes et al [44]	Amount of diabetic patients with controlled blood pressure	Compared to the preimplementation period, more diabetic patients were observed with controlled blood pressure in the postimplementation period (*P*<.001).
	Hawes et al [44]	Amount of diabetic patients with HbA_1c_ levels of less than 8%	Compared to the preimplementation period, more diabetic patients with HbA_1c_ levels of less than 8% were observed in the postimplementation period (*P*<.0001).
	Hawes et al [44]	Amount of diabetic patients with HbA_1c_ levels of less than 7%	Compared to the preimplementation period, more diabetic patients with HbA_1c_ levels of less than 7% were observed in the postimplementation period (*P*<.001).
	Levine et al [45]	Systolic blood pressure	Equivalent outcomes
	Watson et al [38]	Total inflammatory lesion counts	Equivalent outcomes
	Watson et al [38]	Frontal inflammatory lesion counts	Equivalent outcomes
	Watson et al [38]	Leeds score	Equivalent outcomes
	Penza et al [47,51]	Mortality rate	Penza et al reported only descriptive statistics in both studies, so it is unclear if there are differences in mortality rates between e-visits and in-person visits.
	Murray et al [50]; Penza et al [51]	Hospitalizations	Murray et al [50] and Penza et al [51] reported only descriptive statistics, so it is unclear if there are differences in the number of related hospitalizations between e-visits and in-person visits.
	Murray et al [50]	Antibiotic retreatment rate	Equivalent outcomes
	Yokose et al [31]	Proportion of patients serum urate levels of less than 6.0 mg/dL	E-visits had greater proportions of patients with optimal control of serum urate levels when compared to in-person visits (*P*<.01).
**Quality of care**			
	Hawes et al [44]	Amount of diabetic patients receiving aspirin, if clinically indicated	Equivalent outcomes
	Hawes et al [44]	Amount of diabetic patients receiving moderate-intensity statins	Equivalent outcomes
	Hawes et al [44]	Amount of diabetic patients receiving high-intensity statins	Equivalent outcomes
	Heyworth et al [42]	Medication discrepancy discovery rate	It is unclear what the impact is on the rate of discovering medication discrepancies as no *P* value was reported.
	Mehrotra et al [24]	Order rate of diagnostic test	E-visits had a lower order rate of diagnostic tests when compared to in-person visits (*P*<.001).
	Mehrotra et al [24]	Order rate of preventive care services	E-visits had a lower order rate of preventive care services when compared to in-person visits (*P*<.01).
	Yokose et al [31]	Rate that serum urate levels were checked	E-visits had more frequent checks of serum urate levels when compared to in-person visits (*P*<.05).
	Murray et al [50]; Penza et al [46,51]; Mehrotra et al [24]; Courneya et al [40]	Antibiotic prescribing rate	Mehrotra et al [24] reported that the rate of prescribing antibiotics was higher during e-visits than in-person visits for sinusitis (*P*<.001) but not for e-visits for urinary tract infections. However, Penza et al [46] and Murray et al [50] saw equivalent outcomes. Penza et al [51] reported e-visits had lower antibiotic prescribing rates than in-person visits (*P*<.001). Courneya et al [40] also investigated the association but did not report a *P* value, so the impact on antibiotic prescribing rate is unclear.
**Health care utilization**			
	Levine et al [45]	Overall primary care visit utilization	Equivalent outcomes
	Levine et al [45]	Overall specialist visit utilization	Equivalent outcomes
	Levine et al [45]	Overall emergency department utilization	Equivalent outcomes
	Levine et al [45]	Overall inpatient admissions	Equivalent outcomes
	Rajda et al [49]	Number of specialist procedures done 60- and 90-days after initial consultation	E-visits were associated with a lower number of specialist procedures performed 60 and 90 days after an initial consultation when compared to in-person visits (*P*<.01)
	Murray et al [50]; Penza et al [51]	30-day follow-up rate (planned and unplanned)	Equivalent outcomes
	Penza et al [46]; Pathipati et al [43]; Albert et al [39]; Player et al [48]; Adamson et al [19]	Rate of patients who need planned follow-up visits	Penza et al [46] reported that e-visits were associated with higher rates of planned follow-up visits than in-person visits (*P*<.001). Pathipathi et al [43], Albert et al [39], Player et al [48], and Adamson et al [19] reported only the proportion of e-visits that required follow-up visits, so it is unclear what the association of e-visit usage and rate of planned follow-up visits is.
	Penza et al [47]; Mehrotra et al [24]; Courneya et al [40]; North et al [41]; Hertzog et al [30]	Unexpected follow-up encounter rate after initial encounter	Hertzog et al [30] reported that e-visits were associated with higher unexpected follow-up rates when compared to in-person visits (*P*<.05). However, Mehrotra et al [24], Courneya et al [40], and North et al [41] found equivalent outcomes. Penza et al [47] reported only descriptive statistics, so it is unclear if there are differences in unexpected follow-up encounter rates between e-visits and in-person visits.
**Health care costs**			
	Rajda et al [49]; Courneya et al [40]; Rohrer et al [37]	Treatment costs	Courneya et al [40] and Rajda et al [49] reported e-visits were associated with lower treatment costs (*P*<.001). Rohrer et al [37] reported a lower median of costs associated with e-visits than in-person visits (*P*<.01).

^a^HbA_1c_: hemoglobin A_1c_.

### Study Quality Assessment Results

Detailed results of the quality assessment are summarized in Multimedia Appendix 2. Each column describes the quality criterion we assessed. Briefly, most (12/19, 63.2%) studies reported a strategy for minimizing selection bias. Less than half of studies (9/19, 47.4%) reported methods to control for confounders. All studies measured outcomes for e-visits separately from other forms of telemedicine, making it possible to evaluate the unique impact of e-visits on the outcome of interest. Problems with low response rate (<50%) or attrition bias (>10% dropout) were less common (2/19, 10.5%).

### Clinical Outcomes

Associations between e-visit usage and clinical outcomes were reported by 7 studies. A total of 13 different measures were used among the studies. Overall, the studies reported an association with improved outcomes or null findings across the medical conditions examined.

Among diabetic patients, one study (n=36 patients) found that e-visits were associated with significantly improved glucose over a 6-month period (–3.4 percentage points in HbA_1c_, *P<*.001) [44]. In the same study, e-visits for anticoagulant management were associated with less frequent instances of abnormal international normalized ratio values compared to in-person care (5/104, 5% vs 1/198, 0.5%, *P<*.05) [44]. An additional study (n=62) reported that patients with gout who received care through e-visits were more likely to have optimal serum rate levels (>6.0 mg/dL) (63.8% vs 33.9%, *P<*.01) and lower mean serum urate levels (5.5 mg/dL vs 6.7 mg/dL, *P<*.01) compared to historical controls [31]. In the context of acne and hypertension management, equivalent outcomes were reported between e-visits and in-person visits [38,45].

### Quality of Care

Across the 8 studies that examined the association of e-visit usage on quality of care, 8 unique measures were observed. Overall, there were mixed effects on the association of e-visit usage on quality of care.

Mixed results were observed in some quality of care measures of several health care conditions between e-visits and in-person visits [44]. For example, one study (n=36) [44] reported comparable prescribing rates for statins among patients with diabetes across e-visits and in-person visits. One study [24] found that e-visit usage was associated with significantly lower rates of diagnostic procedures for sinusitis (0/475, 0% vs 40/4690, 1%, *P=*.04) and lower rates of diagnostic procedures for urinary tract infections (8/99, 8% vs 1501/2855, 53%, *P<*.001) than those for historical controls. The same study [24] also reported that preventive screenings were lower among sinusitis patients receiving an e-visit (1/475, 0.2% vs 155/4690, 3%; *P<*.001) and urinary tract infection patients’ receiving an e-visit (0/99, 0% vs 214/2855, 7%, *P=*.005) than in those receiving in-person visits.

When examining management of acute infections (eg, urinary tract infections), the definitive impact was less clear. One study [24] found that e-visits resulted in higher antibiotic prescribing rates for sinusitis (471/475, 99% vs 4408/4690, 94%, *P<*.001) but no association for urinary tract infections (98/99, 99% vs 1299/2855, 92%, *P=*.07), compared to those for historical control. Another study [46] reported that e-visits for conjunctivitis resulted in a significantly lower antibiotic prescribing rate than that of phone visits (26/101, 26% vs 84/202, 42%, *P=*.006), and a third study [51] found that e-visits for sinusitis resulted in a significantly lower antibiotic prescribing rate (84/150, 56% vs 108/150, 72%, *P=*.01) than that of in-person visits. Lastly, one study (n=450) [50] reported no difference in antibiotic prescribing rates for treatment of urinary tract infections across e-visits, phone encounters, and in-person visits.

### Access to Care

No studies investigated the association between e-visits and access to care.

### Health Care Utilization

Fourteen studies assessed health care utilization associated with e-visits. An overall mixed impact was observed across these studies.

Some studies evaluated the impact of e-visits on subsequent health care utilization, such as primary care, specialty care, and emergency care [45,49-51]. For example, one study [45] matched 893 e-visits and 893 in-person visits for hypertension and found that e-visits resulted in fewer primary care visits (–0.8 visits, 95% CI 0.3-1.2) compared to in-person visits. The same study [45] also found that usage of specialist visits, emergency department visits, and inpatient admissions were not significantly different across in-person and e-visits. Another study [49] evaluating a teledermatology program found that e-visits were associated with significantly fewer specialty visits at 60-day (15 vs 46, *P=*.005) and 90-day follow-up (26 vs 74, *P=*.001) compared to those associated with in-person visits. Another study [40] examining a direct-to-consumer telemedicine program found that the rate of visits that did not require a follow-up visit (ie, resolution rate) was similar for e-visits for sinusitis (90% vs 91%) and conjunctivitis (94% vs 95%) to those for in-person visits.

Some studies [24,40,41] reported no differences in health care utilization. For example, one study [24] found that e-visits for sinusitis and urinary tract infections had equivalent rates of follow-up visits, phone calls, and emails within 3-week follow-up. Some studies [30,46] reported a higher rate of health care utilization with e-visits. For example, one study [30] found that the rate of follow-up visits was higher for primary care e-visits (59/490, 12%) compared to that for in-person visits (198/2201, 9%; *P=*.04).

### Health Care Costs

Three studies [37,40,49] found e-visit usage was associated with lower overall treatment costs than those for in-person visits. One study utilizing claims data found that e-visits for dermatology resulted in lower mean costs at the initial visit (US $59 vs $113, *P<*.001), at 30-day follow-up ($70 vs $202, *P=*.03), and at 60-day follow-up ($78 vs $221, *P=*.02) than those of in-person visits; however, at the 90-day follow-up, costs were comparable to those of an in-person visit ($86 vs $307, *P=*.08) [49]. One study [40] of a direct-to-consumer e-visit platform reported lower costs for a wide-range of conditions, including sinusitis, conjunctivitis, acne, and ear, nose, and throat infections. As an example, that study (n=9551 visits) used claims data to report that the cost per visit for e-visits was significantly lower for acne management ($178 vs $361, *P<*.001) compared to in-person care [40]. A third study [37] calculated total reimbursable costs and descriptively reported the median cost per visit was lower for e-visits ($161 vs $219) compared to in-person visits for acute conditions (eg, conjunctivitis, sore throat, bronchitis, viral illness, cough).

## Discussion

This was a systematic review that assessed the impact of e-visits on clinical outcomes and health care delivery. To our knowledge, this is the first systematic review to assess the state of evidence for asynchronous e-visits. Most studies found that e-visits were associated with lower treatment costs and comparable clinical outcomes to in-person visits. Studies reported mixed effects on health care utilization, and no studies evaluated the impact of e-visits on health care access, suggesting future research is needed in this area. We provide implications for research and practice below.

Our review found that e-visits may be an adequate substitute for in-person care for chronic disease management. Studies showed that e-visits were effective for the treatment of diabetes, hypertension, and gout. Our findings are consistent with those of previous studies [52] that suggest that other forms of asynchronous communication with providers (eg, secure messaging without a formal e-visit) can improve chronic disease management. Further study, however, is needed to determine the ideal conditions for e-visit implementation. For example, many chronic conditions co-occur with other conditions (eg, diabetes and hypertension), and current studies were not designed to determine whether e-visits are effective for complex patients, such as patients with multiple chronic conditions and older adults who are frail. Additionally, it is important to note that not all types of medical issues may be appropriate for asynchronous management and health care systems may need to implement safeguards to ensure that the right patient is using an e-visit [53]. For example, if patients send a message about an urgent condition, e-visit technology could be harnessed to flag certain keywords (eg, chest pain, breathing difficulties) and display automated pop-up alerts to patients to instruct them to seek care in-person at the office or emergency department [54]. Some health care systems have also implemented guidelines, such as requiring at least one in-person visit prior to an e-visit to ensure that a patient has an established relationship with a clinician [55]. Additional research is needed to determine how e-visits should be implemented (eg, how to prevent inappropriate usage and for which patients does it work best).

Our review found mixed evidence for the effect of e-visits on quality of care. For example, prior studies [22,24-29] have raised concerns that telemedicine usage can increase inappropriate antibiotic prescribing, but this review found mixed evidence on whether comparable antibiotic prescribing rates were observed between e-visits and in-person visits. This inconsistency may stem from differences in the acute infection that was studied (ie, acute sinusitis, urinary tract infections, ear infections, conjunctivitis). Further research is needed to better understand when e-visits can be used effectively for managing acute infections and what implementation strategies can be used to ensure appropriate antibiotic prescribing (eg, use of clinical decision support). Furthermore, additional research is needed to compare the receipt of low-value care (eg, overutilization of services for sinusitis and urinary tract infections across e-visits and in-person visits). Future studies should examine guidelines, such as the Choosing Wisely guidelines [56,57], to see whether e-visits reduce low-value care compared to in-person visits. One study [24] included in this review found that e-visits were associated with lower rates of diagnostic procedures for sinusitis and urinary tract infections and may offer an advantage in terms of reducing unnecessary health care utilization. Studies in this review did report other quality problems, such as lower use of preventive care services in e-visits compared to in-person visits. This may stem from a lack of practice guidance on how e-visits should be implemented (eg, should providers use e-visits as an opportunity to reinforce messages about preventive care?). Prior studies [16] have noted that lack of practice guidance or standard procedures as barriers to e-visit implementation. These differences in quality of care suggest additional implementation research is needed to test implementation strategies for ensuring quality of care delivered through e-visits is consistent across clinicians.

Our review found that e-visits had mixed effects on health care utilization compared to in-person visits. These findings are consistent with those of reviews on other forms of telemedicine [6,58-62]. Specifically, telemedicine can reduce health care utilization in certain instances (eg, reduce the need for in-person visits) or it can increase utilization (eg, meet an unmet demand for a patient that was not previously accessing in-person care). Variation in health care utilization may stem from other various factors that are unaccounted for. For example, if a patient did not submit sufficient information to be evaluated through an e-visit, this could lead to greater health care utilization compared to an initial in-person visit where information exchange is synchronous. Future research is needed to determine strategies for ensuring that complete information is elicited from the patient (eg, structured symptom questionnaires). Additional research should test strategies for optimizing patient data collection and patient-clinician communication through e-visit platforms.

The findings on favorable cost implications align with those of other works that evaluated the effects that synchronous alternatives for care (eg, telemedicine via teleconferencing software) had on cost outcomes for patients [63-70]. Notably, 2 [40,49] of 3 studies used claims data to estimate health care costs; however, insurers commonly include in their contracts with health care organizations contractual adjustments (ie, slightly lower reimbursement rates in exchange for including the health care organization in-network) [71], suggesting that using claims data may underestimate the true cost savings potential of e-visits. Furthermore, many of these studies did not evaluate cost comprehensively (eg, only examined costs of dermatology visits and not visits to providers outside of that delivery system, analyzing average costs per visit instead of average costs of an episode of illness). Additionally, we did not identify any studies that assessed cost-effectiveness, which considers costs relative to outcomes. Future cost-effectiveness studies may also help health care systems make decisions about whether e-visits are worth the investment.

Additional research is also needed on how e-visits impact health care access and the digital divide. There is growing evidence that patient-level disparities exist across adoption and usage patterns of patient portals [72-76]. Similarly, several studies in this review reported differences in usage based on sex [19,30,39,41,42,48], age [30,42,47], employment status [41,48], and ethnicity [19,41]. Since many patients access e-visits through the patient portal, which has known disparities in uptake [72-76], future studies are needed to test strategies for overcoming disparities in patient portal adoption. Since the Pew Research Center reports 81% of Americans in 2019 owned a smartphone [77], there have been studies that have recently tested whether smartphone access to a patient portal could improve access [78,79]. Future studies should test whether strategies, such as smartphone access, could improve uptake of e-visits.

Some of the included literature cited implementation barriers in their discussion that should be further explored in future studies (eg, lack of integration into workflow). Clinicians may need assistance with adjusting their workflows when being trained on e-visits [80]. Best practices should be researched and disseminated to help alleviate concerns on increased workload [81]. Furthermore, the adoption of payment models by insurers that reimburse for e-visits may be a crucial facilitator of e-visit uptake [20,82,83]. Since the COVID-19 pandemic has spurred the introduction of insurance coverage of e-visits among Medicare and some private payers, case studies have been published that suggest e-visits are being used more frequently during the COVID-19 pandemic. For example, one health care system reported that the use of e-visits increased by 4000% during the COVID-19 pandemic and that the majority of e-visits were used for remotely managing patients with COVID-19 [84-86]. Further work is needed to evaluate if the reimbursement policies have led to higher utilization of e-visits by patients and health care organizations.

This systematic review has several limitations. First, a majority of the studies used an observational design, limiting our ability to draw causal conclusions on the effect of using e-visits on quality of care, access to care, costs or clinical outcomes. Second, we found heterogeneity in how studies measured the impact of e-visits, making it impossible to quantitatively pool study estimates. Third, we excluded non-English studies, which may limit our ability to determine the effect of e-visits outside of English-speaking regions. Fourth, the follow-up periods among the studies varied (eg, 2 weeks vs 1 month), limiting our comparisons of findings between studies. Fifth, a majority of the studies examined e-visits that are hosted by the health care delivery organization rather than by a direct-to-consumer vendor or insurer, limiting the generalizability of our results to e-visit programs sponsored by entities external to the health care delivery organization. Lastly, most e-visits occurred in outpatient ambulatory and academic medical center contexts, limiting our ability to comment on inpatient settings.

Overall, the evidence suggests that e-visits can provide equivalent outcomes to in-person care and reduce health care costs for certain health care conditions. There are still notable quality concerns (eg, inappropriate antibiotic prescribing, underutilization of preventive care) that warrant further study. It is also unknown how e-visits have affected access to care. Furthermore, many studies in the review lacked a rigorous design, making it difficult to draw firm conclusions about the state of evidence regarding e-visits. Future trials should be conducted to test the effectiveness of e-visits and determine what factors drive effective implementation.

## Appendix

Multimedia Appendix 1 Search strategy.

Multimedia Appendix 2 Quality assessment of studies.

## Abbreviations

COVID-19: coronavirus disease 2019
e-visit: electronic communication–based visit
