# Supplementary material for: Impact of Asynchronous Electronic Communication–Based Visits on Clinical Outcomes and Health Care Delivery: Systematic Review
Source: J Med Internet Res. 2021 May 5;23(5):e27531. doi: 10.2196/27531 (PMC8135030; doi:10.2196/27531)
Supplement: Multimedia Appendix 1 [file jmir_v23i5e27531_app1.docx]

## **Multimedia Appendix 1: Search strategy**

Database search: We searched the PubMed, Embase, and Web of Science databases for peer-reviewed publications. The search was run on November 2, 2020. We consulted a health sciences librarian to receive feedback on our search terms.

| **Concept** | **MeSH Term or Emtree (Embase subject headings)** | **Additional Search Terms** |
| --- | --- | --- |
| E-visit | None | Evisit  E-visit  Electronic visit  Asynchronous  Virtual visit  Electronic communication  Direct-to-consumer telemedicine  Secure messaging |
| Quality | Quality of health care  Patient satisfaction  Medical Errors  Patient-centered care  Guideline adherence |  |
| Access | Office visits  Waiting lists  Time-to-treatment |  |
| Cost | Health care costs |  |
| Clinical Outcomes | Patient outcome assessment |  |
| Final Search | (evisit OR e-visit OR “electronic visit” OR asynchronous OR “virtual visit” OR “electronic communication” OR “direct-to-consumer telemedicine” OR “secure messaging”) AND (“quality of health care” OR “patient satisfaction” OR “medical errors” OR “patient-centered care” OR “guideline adherence” OR “office visits” OR “waiting lists” OR “time-to-treatment” OR “health care costs” OR “patient outcome assessment”) | |

## 
