# Supplementary material for: Impact of Asynchronous Electronic Communication–Based Visits on Clinical Outcomes and Health Care Delivery: Systematic Review
Source: J Med Internet Res. 2021 May 5;23(5):e27531. doi: 10.2196/27531 (PMC8135030; doi:10.2196/27531)
Supplement: Multimedia Appendix 2 [file jmir_v23i5e27531_app2.docx]

## **Multimedia Appendix 2: Quality assessment of included studies**

| **Citation** | **Study Design** | **Does the study have a strategy for minimizing selection bias, such as a comparison group?** | **Does the study use strategies to control for confounding, such as multivariable analyses and matching?** | **Is the study free of problems with how the exposure was measured (e.g. combining data on e-visits with other types of telemedicine)?** | **Does the study have acceptable response rates (>50%) and/or attrition bias (e.g. dropout rate < 10%)** | **Is information provided about point estimates and errors?** |
| --- | --- | --- | --- | --- | --- | --- |
| Adamson et al., 2010 | Cross- sectional,  observational | No | No | Yes | Yes | No |
| Rohrer et al., 2010 | Cross-  sectional,  observational | Yes | Yes | Yes | Yes | No |
| Watson et al., 2010 | Panel, randomized | Yes | Yes | Yes | No | Yes |
| Albert et al., 2011 | Cross- sectional,  observational | No | Yes | Yes | Yes | No |
| Courneya et al., 2013 | Cross- sectional,  observational | Yes | No | Yes | Yes | No |
| Mehrotra et al., 2013 | Cross- sectional,  observational | Yes | No | Yes | Yes | No |
| North et al., 2013 | Cross-  sectional,  observational | Yes | Yes | Yes | Yes | Yes |
| Heyworth et al., 2014 | Cross- sectional,  case study | No | Yes | Yes | Yes | No |
| Pathipati et al., 2016 | Cross- sectional,  observational | No | No | Yes | Yes | No |
| Hawes et al., 2018 | Panel, Quasi- experimental | No | No | Yes | No | No |
| Levine et al., 2018 | Pooled cross-  sectional, observational | Yes | Yes | Yes | Yes | Yes |
| Penza et al., 2018a | Cross- sectional, observational | Yes | No | Yes | Yes | No |
| Penza et al., 2018b | Cross- sectional, observational | No | No | Yes | Yes | No |
| Player et al., 2018 | Pooled cross- sectional, observational | No | Yes | Yes | Yes | No |
| Rajda et al., 2018 | Panel,  observational | Yes | Yes | Yes | Yes | No |
| Hertzog et al., 2019 | Pooled cross- sectional,  observational | Yes | No | Yes | Yes | No |
| Murray et al., 2020 | Cross- sectional, observational | Yes | No | Yes | Yes | Yes |
| Penza et al., 2020 | Cross- sectional, observational | Yes | No | Yes | Yes | Yes |
| Yokose et al., 2020 | Cross- sectional, quasi- experimental | Yes | Yes | Yes | Yes | Yes |
